# Cytokines and Brain-Derived Neurotrophic Factor as Biomarkers of Cognitive Impairment Related to Breast Cancer and Its Treatments: A Systematic Review

**DOI:** 10.3390/ijms262010074

**Published:** 2025-10-16

**Authors:** Yenny Trinidad Fierro-Salgado, Manuel Reiriz, Ana Isabel Beltrán-Velasco, Javier Calleja-Conde, Xabier Hernández-Oñativia, Sara Uceda, Víctor Echeverry-Alzate

**Affiliations:** 1 NBC Group, School of Life and Nature Sciences, Nebrija University, 28240 Madrid, Spain; yfierros@alumnos.nebrija.es (Y.T.F.-S.); mreiriz@nebrija.es (M.R.); abeltranv@nebrija.es (A.I.B.-V.); jcalleja@nebrija.es (J.C.-C.); xhernandez@nebrija.es (X.H.-O.); suceda@nebrija.es (S.U.); 2 Cardenal Cisneros Higher Education Center, 28006 Madrid, Spain

**Keywords:** BDNF, cytokines, chemobrain, CRCI, cognitive impairment, breast cancer, animal models, biomarkers

## Abstract

Breast cancer is a globally prevalent oncological disease whose treatments, while improving survival rates, often lead to adverse cognitive effects. Brain-derived neurotrophic factor (BDNF) and cytokines, key mediators of the inflammatory response, may play a significant role in these cognitive alterations. This systematic review (osf.io/vk37x) addresses the use of BDNF and cytokines as biomarkers of cognitive impairment in breast cancer animal models. A comprehensive literature search was conducted across the following databases: Web of Science, Scopus, ScienceDirect, PubMed, and Medline. Keywords used were: (“breast cancer” AND “cognitive impairment” AND (“brain derived neurotrophic factor” OR “cytokines”). A total of 9876 articles were identified, of which 17 studies met the inclusion criteria. For quality assessment the SYRCLE’s tool for assessing Risk of Bias was used. Neuroinflammatory and systemic inflammatory responses, particularly increases in pro-inflammatory cytokines (IL-6, IL-1β, TNF-α) and reductions in hippocampal BDNF, are consistently linked to breast cancer and chemotherapy-induced cognitive impairment in animal models. Several interventions normalized these biomarkers and improved cognitive performance after chemotherapy. Anti-inflammatory cytokines (IL-10 or IL-4) were measured in fewer studies and recent research suggests that they could serve as potential protective biomarkers. BDNF, pro- and anti-inflammatory cytokines may represent candidate biomarkers for cancer-related cognitive impairment.

## 1. Introduction

Breast cancer is one of the most prevalent malignancies worldwide and a leading cause of cancer-related mortality among women [1]. Although lung cancer is the primary cause of cancer-related deaths globally, breast cancer continues to impose a significant burden due to its high incidence and profound impact on quality of life [2]. In this sense, in 2022, breast cancer affected around 2.30 million people, representing 11.5% of all cancer types worldwide. Moreover, it is estimated that by 2050, this cancer will affect 3.55 million people globally [3].

Currently, various treatments are available for breast cancer, including surgery, radiotherapy, and neoadjuvant and adjuvant therapies [4,5,6]. To date, chemotherapy remains widely used in both early-stage and locally advanced breast cancer, as it facilitates tumor downstaging, increases the likelihood of breast-conserving surgery, and allows for the assessment of treatment response [7,8].

All these medical advances have led to increased survival of breast cancer patients [9]. In this sense, according to the American Cancer Society [10], since 1989 breast cancer mortality has declined by 44%. The current 5-year relative survival rate is approximately 91% and exceeds 99% for localized-stage diagnoses. However, it is important to highlight that cancer treatments can have a profound impact on patients’ quality of life. Focusing on chemotherapy, it has been reported that, regardless of the specific regimen used, health-related quality of life undergoes significant deterioration [11]. Thus, the main adverse effects observed in patients with breast cancer who have received chemotherapy include somatic symptoms (e.g., dry mouth, gastrointestinal disturbances), psychological symptoms (e.g., stress, anxiety, depression), cognitive impairment, pain, fatigue, and sleep disturbances [12,13].

Regarding the decline in cognitive functions, approximately 65% of patients report this issue during chemotherapy administration, and one in three experiences cognitive deficits one year after the end of treatment [14]. However, according to a recent systematic review, the prevalence of cancer-related cognitive impairment (CRCI) among breast cancer patients varies widely, ranging from 13% to 70%, depending on the type of treatment received and the assessment methods used. This variability reflects the complexity and multifactorial etiology of CRCI, which encompasses biological, psychological, and social factors. Consequently, there is an urgent need for more systematic and harmonized studies to better characterize this phenomenon and to develop effective strategies for its prevention and management within the context of oncological treatment [15]. Cognitive impairments have been measured using different evaluation methods such as standardized neuropsychological tests or self-reports (such as FACT-Cog) and can be associated with structural brain changes observed through neuroimaging techniques [16,17].

In this sense, in the 1980s, the negative cognitive effects observed in women with breast cancer after chemotherapy were referred to as “chemobrain” [15,18]. This term later evolved to chemotherapy-induced cognitive impairment (CICI), and finally to CRCI [19,20]. Moreover, up to 30% of patients experience cognitive impairment even before starting chemotherapy, showing that cancer per se may alter cognitive functions [21]. Despite the use of multiple evaluation approaches, growing evidence suggests that conventional neuropsychological tests may not always be sensitive enough to detect the subtle cognitive deficits reported by patients [22,23]. Discrepancies between objective test results and subjective patient complaints are common, as many patients report cognitive difficulties that are not captured by traditional testing paradigms. This cognitive impairment can have a significant impact on patients’ quality of life, affecting various daily activities such as routine tasks and social interactions [24]. This highlights the need for more refined, ecologically valid, and sensitive cognitive assessment tools that better capture the real-life cognitive difficulties experienced by cancer survivors.

Similarly to clinical observations, preclinical studies using animal models of cancer and chemotherapy have demonstrated a wide range of side effects that mirror those seen in patients. These include neuroinflammation, fatigue, altered sleep–wake cycles, anxiety-like behaviors, and cognitive deficits, even in the absence of tumor burden [25,26,27]. Such models have provided valuable insights into the biological mechanisms underlying CRCI and other quality-of-life disruptions.

Although significant advances in techniques have allowed the detection of specific biomarkers related to breast cancer diagnosis and progression, it is crucial to find new biomarkers related to cognitive impairment throughout the course of the disease that can help improve quality of life [28]. Moreover, since CRCI can appear before, during, or after treatment, biomarkers could help identify the most susceptible patients, allowing for personalized and early intervention. In this context, cytokines and brain-derived neurotrophic factor (BDNF) could serve as biomarkers to detect CRCI in patients with breast cancer [29,30,31].

BDNF is a protein that is widely distributed in the central nervous system and plays a crucial role in neuronal development, differentiation, and survival [32]. BDNF is also fundamental to long-term potentiation (LTP), a key cellular mechanism underlying learning and memory [33,34]. Reduced levels of BDNF have been consistently associated with cognitive dysfunction across various neurological and psychiatric conditions, including depression, Alzheimer’s disease, and aging-related decline [35,36].

Conversely, cytokines are small, non-structural proteins secreted by immune and non-immune cells that mediate and regulate immunity, inflammation, and hematopoiesis. They can be broadly categorized based on function, including interleukins (ILs), tumor necrosis factors (TNFs), and interferons (IFNs), among others. Moreover, cytokines can be divided into pro-inflammatory and anti-inflammatory types, depending on whether they increase or reduce the inflammatory response. This response is a fundamental reaction of the body to any kind of stress, ranging from a simple injury to a complex infection. The initial acute phase of the inflammatory response is multifaceted, involving the synergistic activation of T and B cells. This acute phase is followed by a positive feedback pro-inflammatory loop that is selectively localized to the area of infection or injury. The resolution of acute inflammation is dynamically driven by a tight interplay between anti- and pro-inflammatory cytokines. Major anti-inflammatory cytokines include IL-4, IL-10, IL-11, and IL-13, while IL-1, IL-6, TNF-α, IFN-γ, IL-12, IL-18, and granulocyte-macrophage colony-stimulating factor (GM-CSF) are well-known pro-inflammatory cytokines. It is important to note that transforming growth factor β (TGF-β) exhibits a context-dependent dual role, functioning as primarily anti-inflammatory in acute phases but demonstrating pro-inflammatory activities in chronic disease states. The balance between pro- and anti-inflammatory cytokines is crucial for maintaining immune homeostasis and central nervous system (CNS) integrity. Dysregulation in cytokine signaling can lead to chronic inflammation and has been implicated in neurodegenerative and neuropsychiatric disorders [37,38,39].

In recent years, both cytokines and BDNF have been proposed as potential biomarkers of cognitive impairment in a range of clinical populations [40], including individuals with Alzheimer’s disease [41], Parkinson’s disease [42] and the elderly [43]. Altered BDNF expression and elevated levels of pro-inflammatory cytokines have been observed in patients with cognitive decline, suggesting an interplay between neuroinflammation and impaired neuroplasticity [44,45].

Emerging evidence now indicates that a similar biomarker profile may be observed in cancer survivors, particularly among breast cancer patients undergoing chemotherapy. Studies suggest that chemotherapy can disrupt the balance of pro- and anti-inflammatory cytokines, as well as reduce BDNF expression, which contributes to neuroinflammation and subsequent cognitive impairment [46,47].

Animal models have proven essential not only for replicating behavioral symptoms of CRCI but also for elucidating the underlying biological mechanisms [48]. These models have also shown alterations in key biomarkers such as decreased hippocampal BDNF levels and increased expression of pro-inflammatory cytokines following chemotherapy administration [49,50]. Thus, investigating the relationship between cytokines, BDNF, and cognitive outcomes in cancer survivors represents a promising area of research with potential implications for identifying individuals at risk and developing targeted interventions. Therefore, this systematic review aims to analyze BDNF and/or cytokines as potential biomarkers of cognitive dysfunction associated with breast cancer and its treatments (e.g., chemotherapy), as well as to examine the response of these biomarkers to various interventions intended to mitigate such effects in animal models.

## 2. Materials and Methods

### 2.1. Protocol and Registration

The systematic review protocol was registered on 22 July 2025, with the Open Science Framework (OSF) prior to analysis of the extracted data: https://doi.org/10.17605/OSF.IO/VK37X (accessed on 13 October 2025).

### 2.2. Literature Search

This systematic review followed the Preferred Reporting Items for Systematic Reviews and Meta-Analyses (PRISMA) guidelines [51,52]. We conducted a systematic search for studies published from January 2000 to April 2025. This timeframe was selected because a preliminary scoping search revealed that the majority of preclinical studies examining the interplay between breast cancer, cognitive impairment, and the biomarkers BDNF and cytokines were published after 2000, coinciding with key advancements in the field. The search was performed using five major databases: PubMed, ScienceDirect, Web of Science, Scopus, and MEDLINE. The search strategy included the following terms: “breast cancer” AND “cognitive impairment” AND (“brain derived neurotrophic factor” OR “cytokines”). Boolean operators were used to ensure comprehensive and specific results. Two authors independently conducted the literature search between March and May 2025 (Y.T.F.-S. and V.E.-A.), including the initial screening of titles and abstracts, and the full-text assessment of eligible articles. 

### 2.3. Study Selection

We included studies that met the following criteria: (i) focused exclusively on breast cancer; (ii) used objective and standardized cognitive tests; (iii) measured BDNF and/or cytokines as biomarkers; (iv) were original research articles published between 2000 and 2025 in English and open access format; and (v) the studies involved animals. Exclusion criteria were: (i) studies focused on other cancer types or on mixed cancer populations; (ii) reviews, meta-analyses, case reports, commentaries, or editorials; (iii) studies conducted in humans; and (iv) studies that did not assess cognitive function or did not measure the selected biomarkers. 

**Figure 1:**
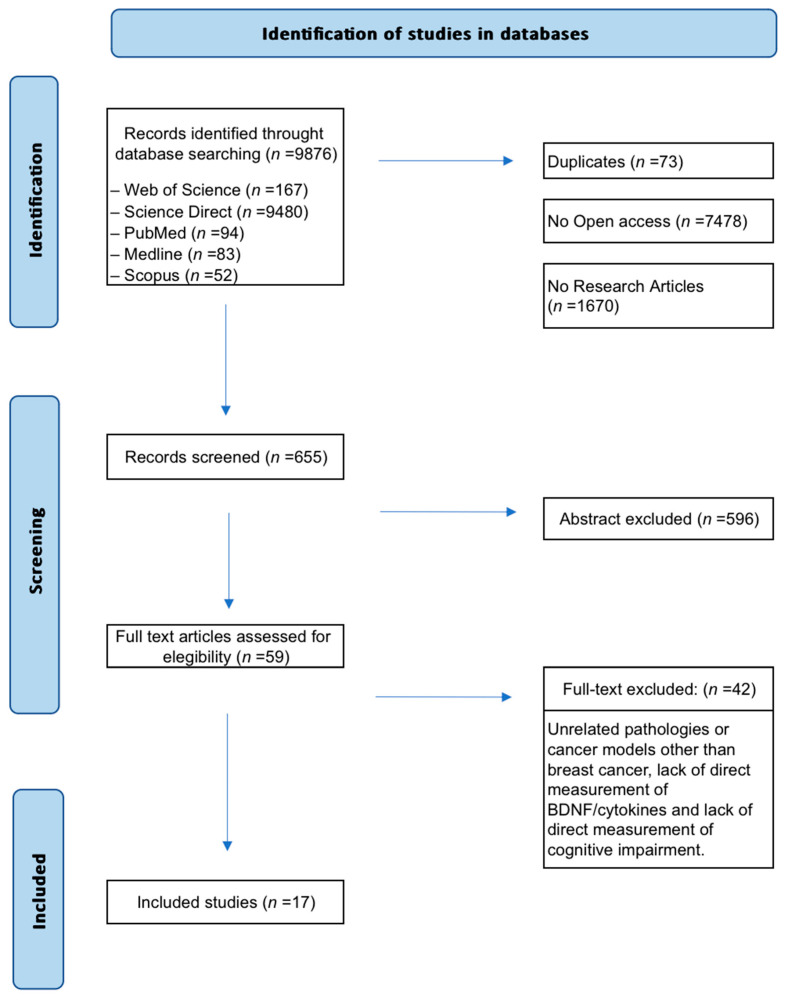
Flow diagram of the search and selection process. Seventeen (*n* = 17) preclinical studies met the eligibility criteria and investigated the role of BDNF and cytokines as biomarkers of cognitive impairment related to breast cancer and its treatments.

A total of 9876 records were initially retrieved (Figure 1). Non-original studies (e.g., reviews, editorials), articles without full-text access, and duplicates were excluded. Then, 655 articles were screened by title and abstract. Of these, 59 met preliminary criteria and underwent full-text review. Forty-two studies were excluded because they were published before 2000, addressed pathologies other than breast cancer, lacked direct measurements of biomarkers such as cytokines or BDNF, or failed to assess cognitive decline. Finally, 17 preclinical studies were included in the synthesis.

## 3. Results

### 3.1. Data Extraction

Once the articles were selected, the included studies were examined for data extraction to conduct a narrative analysis, which also encompassed an assessment of the risk of bias. Data extraction was carried out through the following steps (Table 1):

- Species and strain, and animal model used (i.e., breast cancer, ovariectomy, healthy animals).
- Cancer treatment, dosage and duration.
- Therapeutic interventions aimed at mitigating the tumor/treatment effects, their dosage and duration.
- Cognitive function and paradigm used.
- Analyzed biomarkers (pro- and anti-inflammatory cytokines, BDNF) and biological sample.
- Main findings related to the effects of tumor, cancer treatment, and/or interventions on cognitive functions and biomarkers.

### 3.2. Quality of Included Studies

**Figure 2:**
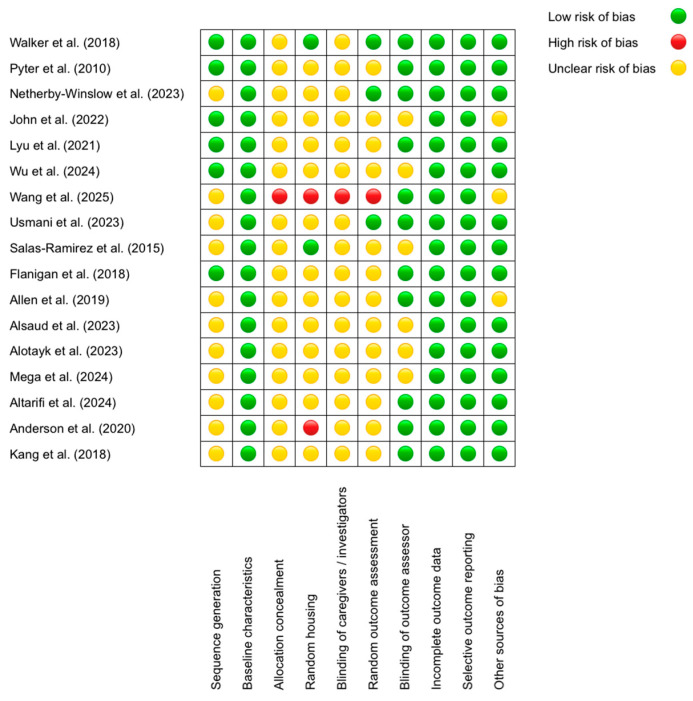
SYRCLE’s tool for assessing Risk of Bias [53,54,55,56,57,58,59,60,61,62,63,64,65,66,67,68,69].

To evaluate the risk of bias in the included studies, we employed the SYRCLE (Systematic Review Center for Laboratory Animal Experimentation) tool, specifically designed to assess methodological quality in animal research. This tool examines 10 key domains, including sequence generation, blinding of outcome assessors, and management of incomplete data, classifying risk as low, unclear, or high (Figure 2).

The results revealed that the domains with the lowest risk of bias were “Baseline characteristics” (all studies received a low rating; e.g., Walker et al. (2018) [53] reported comparable age and sex across groups) and “Selective outcome reporting” (low in 100% of cases, such as in Allen et al. (2019) [63], where all predefined outcomes were reported). Conversely, the domains with the highest risk were “Allocation concealment” (unclear in 16/17 studies; e.g., Alsaud et al. (2023) [64] did not describe concealment methods) and “Blinding of caregivers/investigators” (unclear in 15/17 studies; e.g., John et al. (2022) [56] omitted blinding details). Discrepancies were observed in “Random housing”, with some studies rated as low—for example, Salas-Ramirez et al. (2015) [61] described standardized conditions—and others as high [68], the latter housing animals by treatment group, potentially introducing bias. A recurring pattern was the lack of detailed reporting on randomization and blinding procedures, whereas data completeness and outcome reporting were consistently robust. For instance, Lyu et al. (2021) [57] justified a low risk in “Blinding of outcome assessors” by using automated analysis, while Wang et al. (2025) [59] was rated as having high risk in multiple domains due to methodological omissions. These findings underscore the need for improved documentation of critical procedures to minimize bias in preclinical studies.

### 3.3. Tumor Effects

A total of six out of the seventeen included studies (≈35%) incorporated a study design that allowed for the assessment of the specific effects of tumor growth on biological and cognitive variables [53,54,55,56,57,59]. Among these, two studies [53,54] evaluated these effects exclusively in the absence of any chemotherapeutic intervention. The other four studies [55,56,57,59] first established a baseline in tumor-bearing animals and then proceeded to evaluate the combined effects of subsequent chemotherapy, providing a clear comparison with the treatment impact.

These studies employed both rat and mouse models with different approaches to induce mammary tumors (Table 1). A robust finding across these models was the consistent presence of a systemic (plasma or serum) and hippocampal pro-inflammatory state driven by the tumor. Elevated levels of at least one pro-inflammatory cytokine were reported in tumor-bearing animals compared to healthy controls. Among these, IL-6 emerged as the most consistently and markedly increased marker, suggesting it may be a central mediator of cancer-induced neuroinflammation, followed by IL-1β and TNF-α.

In addition, these studies revealed two distinct patterns of immune response. Three reports [53,55,56] documented a broad pro-inflammatory signature. Walker et al. (2018) [53] demonstrated that soluble factors released by the tumor were sufficient to induce cognitive impairment, as evidenced by their finding that low-dose aspirin, an anti-inflammatory agent, completely prevented cancer-associated cognitive deficits without affecting tumor burden. This supports a causal role for tumor-to-brain inflammatory signaling in cognitive dysfunction. Netherby-Winslow et al. (2023) [55] provided a more detailed mechanism, reporting that the early increase in circulating monocyte chemoattractant protein-1 (MCP-1) in tumor-bearing mice was associated with a sustained increase in activated microglia in the brain. They further proposed that this neuroinflammatory response, together with a significant reduction in hippocampal neurogenesis, underpinned the observed deficits in spatial memory. Finally, John et al. (2022) [56] also found a pro-inflammatory signature in whole-brain samples, although it was not associated with cognitive impairment.

In contrast, a second group of studies reported a more selective inflammatory profile, typically characterized by a significant increase in IL-6 alone or in combination with one other cytokine [57,59]. Pyter et al. (2010) [54], however, found an increase in hippocampal IL-1β, which was the only cytokine assessed in their study.

Regarding cognitive outcomes, findings were inconsistent: while some studies [53,54,55] reported significant impairments, others did not [56,57]. These discrepancies may be attributable to methodological differences, including the specific cancer cell line used, the location and rate of tumor growth, the timing of behavioral testing, and the sensitivity of the cognitive paradigms employed.

Findings on anti-inflammatory cytokines and BDNF were heterogeneous. Three studies measured IL-4 and IL-10 [55,57,59], and only one reported an increase in IL-10 (together with other cytokines) that was associated with memory impairment [55]. Clinical studies in patients with breast cancer before chemotherapy have reported that higher circulating levels of IL-4 and IL-10 are associated with better cognitive performance, particularly in attention and processing speed tasks. These cytokines tend to decrease after chemotherapy, suggesting a potential protective role, especially for IL-4, against chemotherapy-related cognitive impairment [17,70]. However, other studies in early-stage breast cancer have found that impaired cognition is linked to elevated cytokine levels, including IL-4, which may reflect differences in disease stage or timing of sample collection, factors known to influence cytokine profiles [71]. Under physiological conditions, IL-4 functions as an anti-inflammatory cytokine that maintains immune homeostasis, yet within the tumor microenvironment, it can activate the Akt signaling pathway, promoting cancer cell survival. This effect is mediated by STAT6, which induces the expression of anti-apoptotic proteins such as Bcl-2 and Bcl-x, illustrating the context-dependent duality of IL-4 activity [72].

Similarly, IL-10 displays both pro- and anti-tumor properties, depending on the cellular and molecular context. Through its classical anti-inflammatory role, IL-10 can inhibit inflammation, reduce angiogenesis, and activate immune effector cells such as T and NK cells, thereby exerting antitumor effects. Conversely, in metastatic breast cancer cells, IL-10 expression can downregulate cell-mediated immune responses, limit antigen presentation, and enhance immunosuppressive signaling, facilitating tumor progression. This dual behavior reflects an insufficient anti-inflammatory counterbalance in the tumor-driven pro-inflammatory environment, where compensatory increases in IL-4 and IL-10 may become saturated, thereby limiting their regulatory capacity and contributing to cancer-related cognitive dysfunction [73,74].

Regarding BDNF levels, two studies evaluated this marker, and only one reported changes, specifically an increase in brain BDNF, which was not associated with cognitive impairment [56]. The authors hypothesized that this increase could result from long-term microglial activation. This interpretation is plausible, as an increase in microglia-derived BDNF has also been described in different pathological contexts, including breast cancer [75,76]. In such scenarios, microglial BDNF may be associated with neuroinflammatory responses and not necessarily support beneficial synaptic plasticity. However, it is important to highlight that the John et al. [56] study did not distinguish the cellular source of BDNF (neuronal vs. microglial) or investigate the status of its high-affinity receptor, the tropomyosin receptor kinase B (TrkB). This highlights the importance of further investigating the role of BDNF in breast cancer, as previous studies have shown that the expression of its receptor TrkB, particularly the TrkB.T1 isoform, increases tumor resistance to apoptosis. Moreover, BDNF-induced TrkB activation has been implicated in several processes related to tumor progression, including angiogenesis, chemotherapy resistance, invasion, and metastasis [77].

To summarize, tumor-bearing animals consistently showed increased levels of pro-inflammatory cytokines. While IL-6 was the most frequently elevated, only one study reported an association with cognitive impairment [53]. In addition, IL-1β was significantly increased and associated with cognitive impairments in both studies that measured it [54,55]. Findings on anti-inflammatory cytokines (IL-4 and IL-10) were scarce and heterogeneous. The role of BDNF appears complex and context-dependent; although data remain limited, current evidence suggests that its increase in the tumor context may not represent a protective response but rather may reflect prolonged microglial activation, underscoring the need to further investigate its cellular sources and signaling status in the presence of tumors.

### 3.4. Effects of Chemotherapy

A total of 15 preclinical studies included in this review examined alterations in biomarkers and cognitive performance associated with chemotherapy administration. The chemotherapeutic regimens most frequently employed were doxorubicin (DOX), either as monotherapy or in combination with cyclophosphamide (CYP), as well as the CMF regimen (cyclophosphamide + methotrexate + 5-fluorouracil). Other agents such as cisplatin (CIS), 5-fluorouracil (5-FU) alone, and paclitaxel (PAC) were also investigated. Among these, eight studies (53.3%) measured only cytokine levels, including both pro-inflammatory (e.g., IL-1β, IL-6, TNF-α) and anti-inflammatory (e.g., IL-4, IL-10) cytokines [55,57,59,62,63,64,65,66], three studies (20.0%) assessed only BDNF [60,61,67], and four studies (26.7%) assessed both BDNF and cytokines simultaneously [56,58,68,69].

The results of chemotherapy studies reveal a complex interplay between neuroinflammation, neurotrophic disruption, and cognitive impairment, as assessed by various preclinical models. These models provide complementary information: studies in healthy animals isolate the direct neurotoxic effects of chemotherapy agents, while studies in tumor models capture the combined impact of treatment on an already compromised system. A consistent pattern is observed: elevated proinflammatory cytokines and reduced BDNF are central biological factors linked to cognitive dysfunction, although not all agents act through identical mechanisms.

#### 3.4.1. Chemotherapy in Tumor-Bearing Models: Amplifying the Pro-Inflammatory State

In the five studies utilizing tumor-bearing animals [55,56,57,58,59], chemotherapy consistently amplified the pre-existing inflammatory state driven by the tumor. This was characterized by a significant upregulation of key pro-inflammatory cytokines in the hippocampus and periphery. For instance, the regimens involving DOX [57,59] and CMF [56] provoked robust increases in IL-1β, IL-6, and TNF-α. This amplified response was strongly correlated with a marked worsening of cognitive performance in spatial and recognition memory tasks compared to tumor-only controls. The findings suggest that chemotherapy adds a substantial inflammatory burden, likely exceeding the system’s compensatory threshold and resulting in more severe and detectable cognitive dysfunction.

#### 3.4.2. Chemotherapy in Healthy Animals: Diverse Pathways to Neurotoxicity

The eight studies conducted in healthy animals [60,63,64,65,66,67,68,69] confirmed that chemotherapeutic agents are themselves potent inducers of neurotoxicity, though their mechanisms may differ. A clear majority (75%) reported a significant increase in proinflammatory cytokines alongside impairments in working and recognition memory. The most pronounced effects were observed with DOX and 5-FU [65].

However, the study by Kang et al. (2018) [69] provides critical evidence for alternative, non-inflammatory pathways. They reported significant cognitive impairments and structural neuronal damage (reduced dendritic complexity, spine density, and neurogenesis) in the absence of measurable neuroinflammation or microglial activation. As they concluded, “neuroinflammation was not essential for the development of chronic chemotherapy-induced hippocampal dysfunction” in their model [69]. Instead, they proposed that the suppression of key neurotrophic factors like vascular endothelial growth factor (VEGF) and erythropoietin (EPO), leading to neurovascular dysfunction, was a primary mechanism.

This heterogeneity is further highlighted by Alotayk et al. (2023) [65], who found that while DOX and 5-FU significantly increased cytokine levels, CIS and CYP induced cognitive deficits without a significant inflammatory response, suggesting agent-specific mechanisms that may operate independently of cytokine elevation.

#### 3.4.3. Impact of Ovariectomy on Chemotherapy-Induced Neurotoxicity

The effects of chemotherapy in the context of hormonal depletion appear to be model-dependent, revealing a complex interaction rather than simple hormone independence. Salas-Ramirez et al. (2015) [61] reported that cognitive deficits occurred regardless of ovarian status, suggesting a hormone-independent behavioral outcome. However, they found that the molecular mechanisms diverged: ovariectomized rats showed significant chemotherapy-induced alterations in hippocampal signaling pathways, including protein kinase B (Akt) and extracellular signal-regulated kinase (ERK) pathways, and BDNF levels, while intact rats did not. They proposed that ovariectomy induces a vulnerable state that unmasks or potentiates the molecular neurotoxicity of chemotherapy [61].

In contrast, Flanigan et al. (2018) [62] found that ovariectomized mice exhibited attenuated neuroinflammatory and cognitive responses to chemotherapy compared to what is typically observed in intact animals. The authors argued that such discrepancies across studies may be explained by factors such as the dose of DOX and CYP used, as well as the interval between treatment and cytokine measurement. Thus, although a rapid increase in proinflammatory cytokines would be expected based on the clinical literature, hippocampal cytokine levels may have already declined, or soluble cytokine receptors may have been upregulated, by the time behavioral testing was conducted weeks later.

To summarize, although numerous studies have reported the positive effects of ovariectomy on prognosis and survival in breast cancer patients [78,79], the impact of ovariectomy on chemotherapy-induced neurotoxicity has been less explored. However, despite not all studies showing this association [62], some evidence suggests that ovariectomy may unmask or potentiate the neurotoxic and cognitive effects of chemotherapy [61,80], in line with previous studies in ovariectomized animals showing that chemotherapy impairs contextual fear memory [81]. These findings highlight the need for further research to elucidate the mechanisms underlying this interaction and to better understand the role of ovarian hormones in modulating chemotherapy-related neurotoxicity.

#### 3.4.4. BDNF Reduction: A Core but Not Universal Mechanism in Chemotherapy-Induced Neurotoxicity

A significant decrease in hippocampal BDNF was observed in 33.3% (5/15) of the chemotherapy studies; when considering only those that measured BDNF (*n* = 7), the proportion rose to 71.4% (5/7). These involved various regimens, including DOX [58,60,67], CMF [56,68,69], and combined DOX + CYP [69], with one study reporting altered BDNF signaling specifically in ovariectomized rats [61]. This reduction was frequently associated with elevated levels of pro-inflammatory cytokines, primarily IL-1β, IL-6, and TNF-α, and consistently correlated with deficits in cognitive domains such as spatial learning, working memory, and recognition memory. The paradoxical finding of elevated BDNF alongside cognitive impairment in a tumor-bearing model [56] was proposed by the authors to originate from long-term activation of microglial cells rather than representing a beneficial neuroplastic response.

Overall, the studies included in this review suggest that chemotherapy can induce cognitive impairment through multiple pathways. A recurring finding was the elevation of pro-inflammatory cytokines (i.e., IL-1β, IL-6, TNF-α) together with reduced BDNF levels, frequently associated with cognitive deficits in both healthy and tumor-bearing models. Nonetheless, agent-specific differences emerged, as some chemotherapeutic drugs produced cognitive decline without marked neuroinflammation, pointing to the involvement of alternative mechanisms. The influence of hormonal status appeared complex, with behavioral deficits observed in some models regardless of ovarian condition, while the underlying molecular responses diverged.

### 3.5. Effects of the Interventions

Ten preclinical studies reported that pharmacological and natural interventions may mitigate chemotherapy- or tumor-induced neuroinflammation and cognitive impairments. The interventions were categorized by their primary mechanism of action: anti-inflammatory agents, neuroprotective modulators, and multimodal approaches using natural compounds and traditional formulations.

Anti-inflammatory agents attenuated neuroinflammation and improved cognition by targeting specific signaling pathways. The C-C chemokine receptor type 5 (CCR5) antagonist maraviroc was highly effective. The authors proposed that by blocking the C-C motif chemokine ligand 3 and 4 (CCL3/4)-CCR5 axis on microglia, maraviroc inhibited the downstream NF-κB/NLRP3 (nuclear factor kappa-light-chain-enhancer of activated B cells (NF-κB)/NOD-, LRR- and pyrin domain-containing protein 3 (NLRP3)) signaling pathway, leading to a reduction in hippocampal TNF-α and IL-1β levels and the reversal of spatial memory deficits [58]. Similarly, pioglitazone (PIO), a peroxisome proliferator-activated receptor-gamma (PPAR-γ) agonist [64], and low-dose aspirin [53] significantly reduced pro-inflammatory cytokines, with aspirin mitigating cancer-induced cognitive impairment by targeting tumor-derived inflammatory signaling. Allen et al. (2019) [63] found that the colony-stimulating factor 1 receptor (CSF1R) inhibitor PLX5622 effectively depleted microglia. The authors concluded that microglial activation is a major causal factor, as its removal prevented cognitive decline and reduced a broad spectrum of cytokines (IL-1α, IL-1β, IL-3, IL-4, IL-5, GM-CSF) [63]. In a second strategy used by these authors, treatment with human induced pluripotent stem cell-derived microglia (iMG)-derived extracellular vesicle (EV) (iMG-EVs) attenuated microglial activation and restored cognitive function in DOX-treated mice by delivering a bioactive cargo [63].

In contrast, neuroprotective interventions primarily enhanced neuroplasticity by modulating key signaling pathways. The glutamate modulator riluzole significantly restored hippocampal BDNF levels and reversed memory deficits induced by DOX. The authors attributed this effect to the inhibition of glutamate release, preventing excitotoxicity and creating a conducive environment for BDNF expression and neurogenesis [60]. Likewise, the antioxidant vitamin E restored BDNF levels in the hippocampus and improved memory, countering chemotherapy-induced oxidative stress [67]. The pre-treatment with the acetylcholinesterase inhibitor donepezil protected the animals treated with CMF from oxidative damage, reduced the levels of IL-6, IL-1β, and BDNF, and improved spatial learning and memory [56].

Multimodal natural compounds and traditional formulations exerted broad effects on the inflammatory and neurotrophic landscape, demonstrating efficacy in mitigating chemotherapy-induced damage. The flavonoid diosmin (DIOS), tested in healthy rats receiving chemotherapy (DOX), reduced pro-inflammatory markers (i.e., IL-6, IL-1β, TNF-α, MMP-9, COX-2) and enhanced cognitive performance. Moreover, DIOS was found to protect the brain via oxidative stress inhibition, autophagy modulation and down-regulation of apoptotic markers [66]. The multi-herbal extract Mulmina reduced hippocampal inflammation and improved cognitive function, showing comparable efficacy to donepezil. The authors attributed these effects to the combined antioxidant and anti-inflammatory action of its main components (Mangiferin, asiatic acid, and curcumin) [56]. Similarly, in tumor-bearing mice treated with DOX, the traditional formulations Kai-Xin-San (KXS) [57] and Fangxia-Dihuang (FXDH) [59] improved learning and memory, as well as modulated inflammatory markers. Reflecting a holistic approach, their efficacy was attributed to the synergistic action of their components [57,59]. Both formulations reduced pro-inflammatory cytokines (TNF-α, IL-1β, IL-6, IL-12p70) and increased anti-inflammatory cytokines (IL-4, IL-10), with FXDH demonstrating a non-linear dose–response relationship characteristic of complex herbal decoctions [59].

Across categories, the most effective strategies employed a multi-target approach, simultaneously reducing pro-inflammatory signaling, protecting against oxidative stress, and restoring neurotrophic support. The consistent success of these interventions underscores the centrality of neuroinflammation and disrupted plasticity in CRCI pathogenesis and highlights the therapeutic potential of addressing these interrelated pathways.

## 4. Discussion

The main objective of this systematic review was to compile and synthesize the significant findings from animal studies investigating the role of BDNF and/or cytokines as potential biomarkers of cognitive dysfunction associated with breast cancer and its treatments, as well as to evaluate the response of these markers to various interventions aimed at mitigating such effects.

With respect to cancer treatment, this review identified only studies that examined the effects of chemotherapeutic agents in animal models, focusing on changes in biomarkers and cognitive performance in the context of tumor presence and/or chemotherapy. This underscores the need for studies investigating other cancer treatments (e.g., radiotherapy, immunotherapy) and their impact on biomarkers and cognitive functions, to better elucidate treatment-specific effects in this context [82,83].

Regarding cytokines, the findings indicate that significant alterations occur even before the initiation of chemotherapy, suggesting a sustained inflammatory response driven by the presence of the breast tumor itself. Specifically, an early increase in the levels of pro-inflammatory cytokines TNF-α, IL-6 and IL-1β was generally observed across studies (measured in the hippocampus and/or plasma), with these increases being associated with the cognitive impairments observed in the animals. These observations are consistent with previous reports that highlight the tumor’s ability to induce the production of pro-inflammatory cytokines, thereby contributing to neuroinflammatory processes and cognitive dysfunction even in the absence of chemotherapy in women [20]. These cytokines are closely linked to inflammation-associated cancers, where they promote tumor progression and invasion. Their receptor-mediated signaling triggers pro-inflammatory cascades that intensify and sustain the inflammatory response [84,85,86]. For instance, IL-6 plays a central role in inflammation, autoimmunity, cancer, and age-related pathologies, primarily via the IL-6/STAT3 signaling pathway, and elevated levels of TNF-α have been associated with breast cancer recurrence [87,88]. This reinforces the notion that cognitive impairment in breast cancer may arise not only from treatment-related toxicity but also from cancer-induced systemic inflammation.

However, it is important to note that some studies, such as those conducted by John et al. (2022) [56], Lyu et al. (2021) [57], and Wang et al. (2025) [59], did not report significant alterations in cognitive performance in tumor-bearing mice compared to normal controls in certain behavioral tests, despite observing changes in several cytokine levels. These discrepancies may be attributed to methodological differences across studies, including variations in the animal models used or the specific cognitive assessments employed. Similarly, in clinical research, it has been widely reported that conventional neuropsychological tests often lack the sensitivity to detect the subtle cognitive changes described by patients undergoing chemotherapy, which may lead to an underestimation of CRCI in human populations [22,24,89,90]. This parallel limitation highlights the need for more refined and ecologically valid cognitive assessment tools in both preclinical and clinical settings.

In addition, Walker et al. (2018) [53] reported cognitive deficits and elevated IL-6 levels (among other markers), while TNF-α levels were significantly lower in tumor-bearing animals compared to the tumor-free control group. This discrepancy may suggest a more prominent role for IL-6 in tumor-associated inflammation than TNF-α. Alternatively, these divergent findings could be attributed to differences in the murine models, breast cancer cell lines, or tissues analyzed. In line with these findings, Wang et al. (2025) [59] reported an increase in serum IL-6, but did not observe differences in other markers, including TNF-α, or in cognitive performance.

In the context of chemotherapy, the studies analyzed revealed a consistent pattern of increased levels of pro-inflammatory cytokines, particularly IL-1β, IL-6, and TNF-α. In addition, two studies observed a reduction in the levels of anti-inflammatory cytokines (IL-4 and IL-10). In this regard, chemotherapy can stimulate cancer cells to secrete IL-1β, which induces neutrophil extracellular trap (NET) formation, and specific NET-associated proteins are related to chemoresistance [91]. In addition, it is well established that chemotherapy-induced cell death occurs largely through increased oxidative stress by enhancing reactive oxygen species (ROS) and reactive nitrogen species (RNS) production; excessive ROS levels can disrupt the blood–brain barrier (BBB), facilitating the entry of proinflammatory cytokines produced by chemotherapy-targeted tissues [92,93]. Among these, IL-6 has been associated with poor chemotherapy response, in part by regulating autophagy and promoting BECN1 phosphorylation, which strengthens tumor survival under treatment stress [94,95]. TNF-α further contributes to resistance by activating the NF-κB pathway, upregulating inhibitors of apoptosis proteins (IAPs) and other factors [96], and a recent systematic review of studies conducted in breast cancer patients suggests that higher TNF-α levels correlate with reduced chemotherapy efficacy, although more rigorous studies are needed [97]. Together, these findings highlight that IL-1β, IL-6, and TNF-α not only drive chemotherapy-induced inflammatory responses but also actively reinforce resistance mechanisms, underscoring their importance as potential biomarkers and therapeutic targets to improve treatment outcomes.

Our review results showed that these cytokine alterations were associated with impairments in multiple cognitive domains, including learning, spatial and working memory, and to a lesser extent, episodic memory, with the most pronounced effects observed in animals treated with DOX [55,57,58,59,60,61,63,64,65,66]. These effects appeared to be independent of the animal model or the cancer cell line used, suggesting a widespread impact of chemotherapeutic agents on cognitive function through the induction of systemic and neuroinflammation. These findings are supported by clinical studies, which reported that chemotherapy-related cognitive impairment was associated with increased levels of pro-inflammatory cytokines such as TNF-α, IL-6 and IL-1β [29,46,71]. Moreover, a recent systematic review analyzing 15 studies in breast cancer patients receiving chemotherapy reported that IL-6, IL-1β, and TNF-α were associated with varying degrees of cognitive impairment [98]. These clinical findings reinforce our results in animal models, supporting the role of pro-inflammatory cytokines as key contributors to chemotherapy-related cognitive impairment.

Regarding anti-inflammatory cytokines, IL-4 exerts antitumor effects by promoting macrophage and eosinophil infiltration, enhancing the cytotoxic activity of TNF-α, showing additive antiproliferative effects with agents such as TGF-β1 and tamoxifen, and directly inducing apoptosis in breast cancer cell lines [99]. Data also support its potent antitumor activity across various carcinomas, including breast cancer, both in vitro and in vivo [100]. Conversely, IL-10 functions primarily as an immunosuppressive cytokine, inhibiting proinflammatory mediators and IFN-γ expression, thereby fostering an anti-inflammatory tumor microenvironment that promotes immune escape; in human studies, elevated IL-10 levels have generally been linked to poorer prognosis, although its precise role in breast cancer progression remains unclear [101,102,103]. In line with these observations, a recent study in breast cancer patients reported that both IL-4 and IL-10 decreased after chemotherapy, with higher levels of these cytokines showing a protective relationship with attention and processing speed performance [70]. Similarly, in our results, IL-4 and IL-10 decreased following chemotherapy, suggesting that treatment may simultaneously reduce cytokine-mediated antitumor activity and immunosuppressive signaling. Further studies are needed to clarify the role of these cytokines in breast cancer patients undergoing chemotherapy, particularly in the case of IL-10.

The combined DOX + CYP treatment, as well as the CYP and CIS regimens, showed some discrepancies with the general pattern observed. In the study conducted by Alotayk et al. (2023) [65], CYP and CIS did not appear to significantly alter cytokine levels. These differences may be attributed to the use of a single-dose regimen in healthy rats, which may have been insufficient to elicit a detectable inflammatory response in the experimental model employed. Additionally, in the study by Flanigan et al. (2018) [62], the DOX + CYP treatment administered to ovariectomized mice led to few significant behavioral effects and reduced levels of the pro-inflammatory cytokines IL-1β, IL-6, and IL-12 in the hippocampus at the time of assessment. This finding contrasts with the pattern reported in other studies, and the authors suggest that this may be explained by the fact that cytokine levels were measured approximately 10 weeks after the last DOX + CYP administration.

When comparing studies using chemotherapy in healthy versus tumor-bearing animal models, both groups exhibited a consistent pattern of cognitive impairment accompanied by increased levels of pro-inflammatory cytokines, particularly IL-1β, IL-6, and TNF-α. This inflammatory-cognitive association was observed in 80% of tumor-bearing models and 75% of healthy models, suggesting a robust effect of chemotherapeutic agents regardless of tumor presence. Interestingly, Kang et al. (2018) [69], who applied DOX + CYP in healthy mice, reported cognitive deficits despite the absence of cytokine elevation, possibly indicating the involvement of alternative mechanisms such as reduced BDNF. These findings suggest that while neuroinflammation is a common pathway in chemotherapy-induced cognitive dysfunction, its expression and contribution may vary depending on tumor presence, treatment regimen, and molecular pathways involved.

Regarding the role of BDNF, five (71.4%) of the studies that analyzed BDNF reported a significant reduction in BDNF levels in the hippocampus following treatment with cytotoxic agents, which was consistently associated with deficits in spatial learning, working memory, or recognition memory. In four of these studies, chemotherapy was administered to healthy mice [60,68,69] or rats [67], and in one study to tumor-bearing mice [58]. As previously noted, in most of these studies, the reduction in BDNF levels was accompanied by an increase in pro-inflammatory cytokines in the hippocampus, suggesting that chemotherapeutic agents may impair neurogenesis and neuronal survival [56].

In line with our findings in animal studies, chemotherapy-treated patients exhibited reduced BDNF levels. Decreased circulating BDNF has also been reported in neurodegenerative diseases, where it coincides with an upregulation of pro-inflammatory cytokines in the brain, ultimately contributing to neuronal loss. This post-chemotherapy inflammatory state may therefore help to explain the reduction in BDNF levels [35,104].

However, a significant increase in BDNF levels was observed both in tumor-bearing animals and in ovariectomized models. In these studies, BDNF levels remained elevated following treatment with CMF [56] and DOX + CYP [61], respectively, while cognitive impairments persisted. This phenomenon may reflect an adaptive response of the organism to tumor-induced damage and inflammation, or the prolonged activation of microglial cells, which can trigger BDNF release [75,105]. In addition, ovariectomy may induce a generalized stress response that promotes the expression of neuroprotective proteins, such as BDNF, as a compensatory mechanism. However, this stressed state itself may concurrently increase the susceptibility of female subjects to the neurotoxic effects of chemotherapy, despite -or perhaps because of- these compensatory changes [61,80]. Therefore, this remains an area that requires further investigation to elucidate the specific mechanisms underlying hormonal suppression in this context. Notably, some studies in cancer patients have also reported elevated BDNF levels. Through its interaction with a modified form of TrkB, BDNF has been implicated in multiple stages of tumorigenesis, including tumor cell growth, maturation, migration, and invasion. These findings have generated considerable interest in exploring BDNF as a potential diagnostic and prognostic biomarker for cancer [76,106].

Ten preclinical studies exploring the effects of therapeutic interventions on neuroinflammation and cognitive deficits induced by tumor or chemotherapy, were included in this systematic review. These pharmacological compounds, natural agents, and multi-component traditional formulations consistently led to a reduction in pro-inflammatory cytokines (mainly TNF-α, IL-1β, IL-6, and IL-12p70), promoted the restoration or increase in hippocampal BDNF, and were associated with improved performance in several cognitive tasks.

Fifty percent of these studies were conducted using chemotherapy in healthy animals [60,63,64,66,67], while 40% applied chemotherapy in tumor-bearing animal models [56,57,58,59]. One study [53] administered low-dose aspirin to tumor-bearing mice without chemotherapy. This highlights the potential of these interventions to counteract the negative effects of cytotoxic agents, whether in the presence or absence of tumor.

However, it is noteworthy that there was only one study for each type of compound investigated (e.g., riluzole, mulmina, donepezil, maraviroc, or pioglitazone), and doxorubicin was the chemotherapeutic agent used in 80% of these studies, indicating a strong focus on anthracycline-induced neurotoxicity in the current preclinical literature [107]. Two of the reviewed studies used paclitaxel (PAC) or CMF (cyclophosphamide + methotrexate + 5-fluorouracil); however, further research is needed on interventions that may have a neuroprotective role against these and other chemotherapeutic classes or regimens, which will enhance the generalizability of the findings and provide relevant insights into the role of cytokines and BDNF as potential biomarkers of cancer-related cognitive impairment (CRCI).

A recent review analyzing both animal and human studies suggests that a range of interventions may help mitigate CRCI, potentially through mechanisms associated with changes in underlying biomarkers. Anti-inflammatory agents, antioxidants, and mitochondrial-supporting therapies have been reported to preserve neuronal metabolism and reduce neuroinflammation, while hormonal management may protect against chemotherapy-induced menopause, an additional factor contributing to cognitive decline in breast cancer patients. Similarly, modulation of gut microbiota through probiotics or prebiotics can restore microbial diversity and reduce neuroinflammatory processes, and cognitive rehabilitation strategies such as mindfulness practices, behavioral therapy, and physical exercise support neuroplasticity [108]. Focusing on human studies, Tong et al. (2018) [109] reported that acupuncture therapy is effective in treating CRCI in breast cancer patients, possibly through an increase in BDNF. Other clinical studies also report cognitive improvements following both pharmacological and non-pharmacological interventions, including Tibetan sound meditation, dexmethylphenidate, modafinil, donepezil, brief cognitive-behavioral therapy, cognitive training targeting executive functions, and aerobic exercise [110,111,112,113,114,115,116]. However, these studies do not analyze the relationship between such interventions and biomarker changes, highlighting the need for future clinical research that systematically addresses this aspect.

Moreover, there is evidence of cognitive impairment after chemotherapy similar to the effects after irradiation, and after combined treatment (multimodal regimens) [117]. Nevertheless, the preclinical studies included in this review exclusively employed chemotherapeutic agents, underscoring the need to investigate other treatment modalities—both in breast cancer and in other cancer types—as well as additional therapeutic strategies to prevent or treat CRCI in these contexts.

These preclinical findings reinforce the notion that neuroinflammatory and systemic inflammatory responses, particularly elevated levels of pro-inflammatory cytokines IL-6, IL-1β, and TNF-α, together with reductions in hippocampal BDNF, are consistently linked to cognitive impairment in animal models of breast cancer and chemotherapy-induced cognitive impairment. Notably, while anti-inflammatory cytokines such as IL-10 and IL-4 were measured in fewer studies, recent research suggests that they could serve as potential protective biomarkers [118].

From a clinical perspective, CRCI remains a major concern for cancer survivors, affecting quality of life, daily functioning, and treatment adherence. Using BDNF combined with pro- and anti-inflammatory cytokines could enhance early detection and risk stratification in clinical settings. Indeed, analogous studies in other medical fields have shown that combining pro-inflammatory cytokines (e.g., IL-6, IL-1β, TNF-α) with additional biomarkers significantly improves diagnostic accuracy [119,120]. It is plausible that a similar biomarker panel, linking BDNF and cytokines, could offer superior predictive utility for CRCI. However, significant gaps remain. Most studies focus on a few chemotherapeutic agents, primarily doxorubicin. Clinical translation requires inclusion of broader regimens, combinatorial therapies, and consideration of anti-inflammatory mediators such as IL-10 and IL-4.

In summary, the preclinical data reviewed support the hypothesis that proinflammatory cytokines (IL-6, IL-1β, TNF-α) and BDNF are functionally interlinked in CRCI. Anti-inflammatory cytokines represent an underexplored but potentially key component as biomarkers. Future research should focus on validating biomarker panels (cytokines + BDNF) in longitudinal human studies, optimizing both diagnosis and personalized interventions to protect cognitive health in cancer survivors.

## 5. Study Limitations

While this systematic review provides a comprehensive analysis of the selected biomarkers, several considerations regarding its scope and methodology should be acknowledged.

Firstly, the analytical focus was deliberately placed on BDNF and cytokines. This choice was based on their established relevance and methodological consistency in the existing literature, which enabled a coherent synthesis. We acknowledge, however, that this narrow scope excludes the potential contribution of other immune mediators and alternative pathological mechanisms. Although necessary for a detailed examination, this focus does not negate the multifactorial nature of cognitive impairment and highlights an important avenue for future research.

Secondly, to ensure full transparency and replicability of the screening process, this review relied exclusively on open-access literature. While this pragmatic decision facilitated access, it may also have introduced selection bias by excluding potentially relevant studies published in subscription-based journals. Future reviews should therefore aim to integrate a broader range of sources to further strengthen the evidence base.

Thirdly, the interpretative power of any systematic review depends on the reporting quality of the included primary studies. A key challenge encountered was the incomplete description of methodological details, such as blinding and randomization procedures, in some studies. These reporting gaps inevitably limit the depth of risk-of-bias assessments that can be performed and should be taken into account when interpreting the conclusions presented here.

Finally, another important consideration relates to the translational value of the biomarkers assessed. Since this systematic review is based on animal models, access to brain tissue represents a strength, and several studies included analyses in the hippocampus or whole brain (while others used plasma or serum). However, for clinical applicability, biomarkers should ideally be measurable in accessible biological fluids such as blood or saliva. This requires further confirmation in animal studies incorporating plasma or serum measurements, as well as in clinical trials designed to evaluate cytokines and BDNF in comparable sample types.

Taken together, these limitations highlight the need for more rigorous and transparently reported preclinical research, as well as complementary investigations into additional biomarkers, to advance our understanding of cancer-related cognitive impairment.

## 6. Conclusions

This systematic review highlights consistent associations between increased pro-inflammatory cytokines (i.e., IL-6, IL-1β, TNF-α), reduced levels of hippocampal BDNF, and cognitive impairment in preclinical models of breast cancer and chemotherapy. Overall, these findings underscore the complex role of pro-inflammatory and anti-inflammatory cytokines, as well as BDNF, as potential biomarkers in cancer and chemotherapy outcomes. Pro-inflammatory cytokines such as IL-1β, IL-6, and TNF-α not only drive tumor progression and invasion but also contribute to chemotherapy resistance, underscoring their relevance as both biomarkers and therapeutic targets. Conversely, the decrease in IL-4 and IL-10 observed after chemotherapy suggests that treatment may reduce both cytokine-mediated antitumor effects and immunosuppressive signaling, although further studies are particularly needed to clarify the role of IL-10 in this context. With respect to BDNF, while our results in animals, as well as some clinical studies, show reduced levels after chemotherapy and its association with cognitive decline, other reports indicate increased levels in cancer patients, where BDNF may promote tumor growth, maturation, and invasion.

Importantly, several interventions (pharmacological, naturally derived, and traditional formulations) reduced neuroinflammation, normalized BDNF levels, and improved cognitive performance, both in tumor-bearing and healthy chemotherapy-treated animals. The evidence suggests that combining cytokine profiles with BDNF measurements could improve early identification of cancer-related cognitive impairment and guide targeted interventions. Future studies should validate these findings in clinical contexts, explore additional treatment regimens, and assess biomarker panels to support personalized strategies for preserving cognitive function in cancer survivors.

## Figures and Tables

**Table 1:** Effects of tumor presence, cancer treatments, and/or interventions on cognitive functions and biomarkers.

Strain/Model	Cancer Treatment (Dosage and Duration)	Intervention (Dosage and Duration)	Cognitive Function: Paradigm	Biomarker (Biological Sample)	Results	References	
Female BALB/c and C57BL/6J mice/Orthotopic implantation of mammary adenocarcinoma tumor cells (4T1.2 or EO771, respectively).		Aspirin (25 mg/kg/day for 2 weeks)	Episodic memory: NOR. Spatial memory: NPR	IL-1α, IL-6, MIP-1α, G-CSF, TNF-α and CXCL1 (Plasma)	Tumor-bearing mice exhibited increased plasma levels of IL-1α, IL-6, MIP-1α, G-CSF and decreased levels of TNF-α and CXCL1. These changes coincided with the development of NOR and NPR memory deficits between days 4 and 7. Low-dose aspirin (oral) completely prevented tumor-induced memory impairments without affecting tumor burden or locomotion.	[53]	10.1371/journal.pone.0208593
Wistar female rats/N-nitroso-N-methylurea (NMU)-induced mammary tumor model			Episodic memory: NOR. Spatial learning and working memory: MWM. Reference and working Memory: RAM	BDNF and IL-1β (Hippocampus)	Rats with tumors showed increased hippocampal gene expression of IL-1β, with no significant changes in BDNF levels observed. Cognitively, they showed impaired spatial reference memory (RAM) and were unable to recognize new objects (NOR), while working memory and performance in the MWM were unaffected.	[54]	10.1016/j.bbi.2010.02.004
Female C57BL/6 mice/Orthotopic implantation of mammary adenocarcinoma tumor cells.	DOX (10 mg/kg) + CYP (200 mg/kg) (once a week for 4 weeks)		Spatial learning and memory: Delayed spatial alternation	TNF-α, IL-1β, IL-2, MCP-1, IL-10, IL-6, IL-17, IL-4 (Serum)	The presence of tumor was associated with elevated serum concentrations of TNF-α, IL-1β, IL-2, MCP-1, and IL-10, which correlated with impairments in delayed spatial alternation memory. Treatment with chemotherapy (DOX + CYP) further amplified inflammatory response and worsened spatial memory deficits.	[55]	10.1016/j.bbih.2023.100699
Female BALB/c mice/Orthotopic implantation of TNBC tumor cells (4T1)	CMF: CYP (50 mg/kg) + MTX (5 mg/kg) + 5-FU (50 mg/kg) (once a week for 3 weeks)	Mulmina MN (40 mL/kg and 80 mL/kg) and Donepezil DPL: (2 mg/kg). Daily oral treatment of MN and DPL began one week before CMF.	Spatial learning and memory: MWM	BDNF and TNF-α, IL-1β, IL-6, MIP-1α (Brain)	In tumor-bearing mice, hippocampal levels of IL-1β, IL-6, TNF-α and BDNF were elevated. The presence of tumor did not cause significant cognitive impairment. Administration of CMF was associated with a further increase in cytokine levels and induction of cognitive deficits. Co-treatment with both MN and DPL significantly reduced IL-1β, IL-6, and BDNF levels, and improved cognitive function compared to CMF alone.	[56]	10.1038/s41598-022-06862-9
Female BALB/c mice/Orthotopic implantation of TNBC tumor cells (4T1).	DOX (5 mg/kg once a week for 3 weeks)	Kai-Xin-San KXS: (1.5 g/kg for 21 consecutive days).	Spatial learning and working memory: MWM	IL-1β, IL-6, TNF-α, IL-12p70, IL-4, and IL-10 (Hippocampus and Serum)	The presence of tumor induced a mild elevation in serum levels of IL-6 and IL-12p70 but did not cause cognitive impairment. DOX treatment significantly worsened spatial memory, amplified hippocampal levels of IL-6, IL-12p70, TNF-α, IL-1β, and suppressed the anti-inflammatory activity of IL-4 and IL-10. Intervention with KXS reversed cognitive deficits, reduced pro-inflammatory cytokine levels, and restored the balance of IL-4 and IL-10.	[57]	10.1155/2021/5521739
Female C57BL/6J mice/Orthotopic implantation of E0771 TNBC tumor cells.	DOX (5 mg/kg once a week for 4 weeks)	Maraviroc MVC: (10 mg/kg, five times a week for 4 weeks)	Spatial learning and working memory: MWM	BDNF and IL-1β, TNF-α, IL-6, CCL3, CCL4 (Hippocampus)	Treatment with DOX resulted in increased mRNA expression and protein levels of the chemokines CCL3 and CCL4, as well as increased protein levels of IL-1β and TNF-α in the hippocampus. DOX treatment also decreased BDNF levels and induced spatial memory impairment. Administration of MVC attenuated the DOX-induced neuroinflammation and systemic inflammation, restored hippocampal BDNF levels, and improved cognitive performance.	[58]	10.1016/j.neuropharm.2024.109981
Female BALB/c mice/Orthotopic implantation of TNBC tumor cells (4T1)	DOX (5 mg/kg once a week for 3 weeks)	Fangxia-Dihuang Decoction FXDH (0.28 g/mL, 0.56 g/mL, 0.78 g/mL daily for 3 weeks)	Episodic memory: NOR. Working memory: Y-Maze	TNF-α, IL-12p70, IL-4, IL-10, IL-6 (Serum and hippocampus)	Tumor-bearing mice showed elevated serum levels of IL-6 and mild, non-significant, cognitive impairments. DOX treatment increased the levels of IL-6, IL-12p70, TNF-α while reducing the levels of IL-10 and IL-4; it also impaired working and recognition memory. Treatment with FXDH resulted in reduced levels of pro-inflammatory cytokines, increased levels of anti-inflammatory cytokines, and improved cognitive performance.	[59]	10.3389/fonc.2025.1515498
Female C57BL/6J wild-type mice/Chemotherapy applied in healthy mice.	DOX (2 mg/kg once a week for 4 weeks)	Riluzole RZ: (13 mg/kg for 30 days).	Spatial memory: NPR. Fear extinction memory: FE	BDNF (Hippocampus)	DOX treatment resulted in ~45% reduction in hippocampal BDNF levels and impaired spatial and recognition memory. Administration of RZ restored BDNF levels and reversed cognitive deficits.	[60]	10.1007/s13311-022-01339-z
Female Sprague-Dawley rats/Chemotherapy in healthy and ovariectomized rats.	DOX (4 mg/kg) + CYP (40 mg/kg) (once a week for 3 weeks)		Working memory: Y-Maze. Episodic and spatial Memory: NOR	BDNF (Hippocampus)	Treatment with DOX + CYP did not alter hippocampal BDNF levels, while ovariectomized animals showed higher BDNF levels than intact controls regardless of chemotherapy. Chemotherapy was associated with significant cognitive deficits (spatial and working memory). Ovariectomy did not affect the severity of these cognitive impairments.	[61]	10.1016/j.bbr.2015.06.028
Female C57BL/6J mice/Chemotherapy to ovariectomized mice.	DOX (2 mg/kg) and/or CYP (50 mg/kg) (once a week for 4 weeks)		Learning and spatial memory: MWM. Spatial Memory: NPR. Episodic Memory: NOR	IL-1α, IL-1β, IL-2 to IL-6, IL-10, IL-12p70, IL-17, MCP-1, IFN-γ, TNF-α, MIP-1α, RANTES (Hippocampus)	Levels of IL-1β, IL-6, and IL-12 in DOX + CYP-treated mice were significantly decreased compared with saline treated mice. Density of stubby spines, but not mushroom or thin spines, in the dentate gyrus was significantly decreased in the DOX, CYP, and DOX + CYP groups. These treatments were not associated with long-term cognitive impairment.	[62]	10.1093/toxsci/kfx267
Male wild-type C57BL/6 mice/Chemotherapy applied in healthy mice.	DOX (2 mg/kg once a week for 4 weeks)	1. Inhibition of CSF1R (PLX5622) 2. iMG-EV	Episodic memory: NOR. Spatial memory: NPR. Fear memory consolidation: FC	Cytokine gene expression: IL-1α, IL-1β, IL-3, IL-4, IL-5, IL-12, GM-CSF, RANTES, MIP-1α and CCL5 (Hippocampus)	DOX treatment resulted in significantly increased levels of IL-1β, IL-3, IL-5, IL-12, and GM-CSF, and impaired memory. Co-treatment with PLX5622 was associated with restored cognitive performance, normalization of IL-1α, IL-1β, IL-3, IL-4, IL-5, and GM-CSF levels, and increased RANTES and MIP-1α levels. Treatment with iMG-EVs also led to a complete restoration of cognitive performance and attenuated microglial activation.	[63]	10.1186/s40478-019-0838-8
Wistar female rats/Chemotherapy applied in healthy rats.	DOX (5 mg/kg twice a week for 14 days)	Pioglitazone PIO: (2 mg/kg, twice per week for 14 days)	Episodic memory: NOR. Working memory: Y-Maze	IL-6, IL-1β, and TNF-α (Brain)	DOX treatment increased levels of IL-1β, TNF-α, and IL-6 and impaired cognitive performance in both Y-Maze and NOR. Co-treatment with PIO significantly reduced TNF-α and IL-6 levels and normalized Y-Maze performance (non-significant improvement in NOR).	[64]	10.3390/molecules28124775
Wistar female rats/Chemotherapy applied in healthy rats.	DOX (25 mg/kg), CYP (200 mg/kg), 5-FU (100 mg/kg), CIS (8 mg/kg) (single dose)		Episodic memory: NOR. Working memory: Y-Maze	TNF-α, IL-1β, IL-6 (Hippocampus)	DOX treatment increased TNF-α, IL-1β and IL-6 levels, and was associated with impairments in spatial learning and memory, recognition of new objects, and spontaneous alternation. CYP treatment also elevated TNF-α and IL-1β levels, but to a lesser extent, with no significant changes in IL-6, and induced mild cognitive deficits in spatial and working memory. 5-FU treatment increased TNF-α and IL-6 levels, but not IL-1β levels, impaired spatial learning and recognition of new objects. Treatment with CIS appeared to impair short-term memory, with no effects in TNF-α, IL-1β, and IL-6.	[65]	10.1016/j.biopha.2023.115245
Male Wistar rats/Chemotherapy applied in healthy rats.	DOX (3 mg/kg once weekly for 56 days)	Diosmin DIOS: (40 mg/kg daily for 56 days)	Episodic memory: NOR. Working memory: Y-Maze	IL-6, IL-1β, TNF-α, MMP-9 and COX-2 (Brain)	Treatment with DOX elevated the levels of IL-6, IL-1β, TNF-α, MMP-9 and COX-2, and impaired episodic memory. Co-treatment with DIOS largely reversed the cytokine elevations and fully restored cognitive performance in the NOR task and Y-maze.	[66]	10.1016/j.dscb.2023.100111
Female Sprague-Dawley rats/Chemotherapy applied in healthy rats.	DOX (2 mg/kg/week) or PAC (2 mg/kg, every other day) (4 weeks)	Vitamin E α-tocoferol: (100 mg/kg daily for 4 weeks)	Learning and memory: Radial arm water maze (RAWM)	BDNF (Hippocampus)	Treatment with DOX and PAC decreased BDNF levels and resulted in short-term memory impairment. Co-administration of vitamin E restored BDNF levels and improved cognitive outcomes.	[67]	10.1007/s00280-023-04602-y
Male C57BL/6J mice/Chemotherapy applied in healthy mice.	CMF: CYP (100 mg/kg) + MTX (10 mg/kg) + 5-FU (100 mg/kg) (once a week for 4 weeks)		Episodic memory: NOR. Working memory: Y-Maze	BDNF and IL-1α, IL-1β, IL-3, IL-10, TNF-α (Hippocampus)	CMF treatment resulted in decreased BDNF levels, increased levels of pro-inflammatory cytokines (IL-1α, IL-1β, IL-3, IL-10, and TNF-α), and deficits in spatial working memory and recognition memory.	[68]	10.1093/toxsci/kfz213
Female C57BL/6 mice/Chemotherapy applied in healthy mice.	DOX (2 mg/kg) + CYP (50 mg/kg) (once per week for 4 weeks).		Working memory: Y-Maze. Episodic memory: NOR	BDNF and TNF-α, IL-1β, IL-6 (Hippocampus)	Treatment with DOX + CYP did not increase pro-inflammatory cytokine levels, but it significantly reduced BDNF protein levels. Treated mice showed impaired recognition memory (NOR task) compared to controls.	[69]	10.5607/en.2018.27.5.419

## Data Availability

Not applicable.
